# Five-year fracture risk assessment in postmenopausal women, using both the POL-RISK calculator and the Garvan nomogram: the Silesia Osteo Active Study

**DOI:** 10.1007/s11657-021-00881-1

**Published:** 2021-02-16

**Authors:** Piotr Zagórski, Elżbieta Tabor, Katarzyna Martela-Tomaszek, Piotr Adamczyk, Wojciech Pluskiewicz

**Affiliations:** 1 Department of Orthopaedic Surgery, Sports-Clinic, Żory, Poland; 2 Department and Clinic of Internal Diseases, Diabetology, and Nephrology, Faculty of Medical Sciences in Zabrze, Medical University of Silesia, Katowice, Poland; 3 Silesian Academy of Medical Sciences, Katowice, Poland; 4 Department of Paediatrics, Faculty of Medical Sciences in Katowice, Medical University of Silesia, Katowice, Poland; 5 Department and Clinic of Internal Diseases, Diabetology, and Nephrology, Metabolic Bone Diseases Unit, Faculty of Medical Sciences in Zabrze, Medical University of Silesia, Katowice, Poland

**Keywords:** Concordance, Fracture risk, Garvan, POL-RISK

## Abstract

***Summary*:**

The study project was designed to assess the concordance of clinical results in the assessment of 5-year fracture risk of any fracture, carried out by two methods: the Garvan algorithm and the POL-RISK model. The study group included 389 postmenopausal women of Caucasian race. The concordance of results, obtained by those two models, turned out to be moderate, and the threshold for high fracture risk group was 11% in the POL-RISK model.

**Purpose:**

The goal of the study was to evaluate the concordance of results in fracture risk assessments between the Garvan Fracture Risk Calculator and POL-RISK, a new Polish algorithm, and to define an optimal threshold for intervention.

**Methods:**

The study was a part of the Silesia Osteo Active Study. A group of 389 postmenopausal women, aged 65.2±6.9 years (mean ± SD), was randomly selected from the general population of Zabrze, Poland. All the participants had bone densitometry examination to assess the bone mineral density of the femoral neck. The mean femoral neck *T*-score was (−0.99) ± 1.05 SD. 6.4% of the women revealed osteoporosis. Five-year risk of any fracture was assessed, using the Garvan and POL-RISK calculators. The performance of each model was evaluated by the area under the receiver operating characteristic curve (AUC).

**Results:**

The median 5-year risk of any fracture was 7% (range 1–54%) in the Garvan model and 8.8% (range 1.1–45.5%) in the POL-RISK algorithm. There was a significant correlation between the results obtained by both methods (*r*=0.6, *p*<0.005). For the thresholds, assumed at 8% and 13% (according to recommendation derived from Garvan tool), the rates of concordance of results between both calculators were 76% and 84%, respectively. In ROC analysis for the POL-RISK method, performed with reference to the Garvan method at two different cut-offs, assumed to be high fracture risk indicators (8% and 13%), the AUC values were 0.865 and 0.884, respectively. The optimal threshold for high fracture risk in the POL-RISK algorithm was ≥ 11%, which yielded a sensitivity of 0.94 and a specificity of 0.71.

**Conclusion:**

The obtained data demonstrate a moderate concordance of results between the POL-RISK algorithm and the Garvan model, illustrated by low and high fracture risk cut-offs, established in ROC analysis. In addition, the threshold of 11% in the POL-RISK method was the optimal level for “high risk”.

## Introduction

Osteoporosis is a common disease, often underdiagnosed in the elderly population. This medical condition is defined as generalized bone weakening, leading to an increased risk of fragility fracture [1]. Postmenopausal women—in whom the lack of protective oestrogens accelerates bone resorption—are the major group at risk of osteoporosis [2, 3]. Osteoporotic fractures become a large individual and social burden—elderly patients with hip or vertebral fractures suffer from pain, often from lifelong immobilization and its thrombotic and inflammatory complications which, in general, increase the mortality rate among the patients [4, 5]. The medical costs of osteoporosis treatment (including post-fracture treatment and pharmacological interventions) are prognosed to increase in the European Union by 25% between 2010 and 2025 [6].

Nowadays, the gold standard in osteoporosis diagnostics is dual-energy X-ray absorptiometry (DXA) of the central skeleton—lumbar spine and proximal femur [7–9]. Bone mineral density (BMD) was measured by DXA and expressed in *T*-score standard deviation values in relation to young, healthy women, 20–29 years old.

As a clinical decision should not be based on raw measurement results only, a question was raised who and when should be offered the treatment first. The FRAX calculator was proposed to help solve the problem [10]. It was first launched in 2008 in 8 countries [11] and nowadays, it has already been validated in 64 countries, including Poland [12]. This computer-based algorithm assesses the 10-year probability of hip and major fractures. The calculator has become popular worldwide as the first tool, combining DXA results with clinical factors. Nevertheless, it has still some limitations, such as discounting of dose-responses, regarding several important risk factors or not taking into account the history of falls or other co-morbidities. The tool also offers only the 10-year risk calculation [13]. As in response to those unsolved problems, new fracture risk assessment tools were designed. One of them was developed by the Garvan Institute of Medical Research to evaluate 5- and 10-year risk of hip fracture or any fracture [14, 15]. Unfortunately, it has no validation to be used in the Polish population.

A Polish algorithm, called POL-RISK (based on RAC-OST-POL Study) [16], was first launched in a Polish postmenopausal population in 2017 [17, 18]. This calculator was designed to help clinicians assess the risk of any fracture in a 5-year perspective. As it still has no optimal cut-offs for therapeutic decision, its use is somewhat limited. Therefore, the aim of this study was to analyse the conformity between POL-RISK and Garvan Tool models in the aspect of a 5-year fracture risk assessment and to find the optimal thresholds in the POL-RISK algorithm for medical intervention.

## Methods

### Subjects

The presented study was a part of the epidemiological research project, called the Silesia Osteo Active Study [19, 20]. The participants were 389 postmenopausal Caucasian women in the mean age of 65.2 ± 6.9 years (range 55–87 years) who responded to an invitation, sent by regular mail to randomly selected citizens of Zabrze, aged over 55 years. The study obtained positive Bioethical Committee opinion (KNW/0022/KB1/22/14). All the participants of the study signed an informed consent form prior to investigation. Then, all of them filled out self-reported questionnaires, regarding co-morbidities (important in exclusion of pathological fractures resulting e.g. from oncological conditions) and fracture history (the age of fracture occurrence, localization, circumstances, the use of glucocorticosteroids (with trade/international names, doses and the route of administration)). The main risk factors, such as prior fractures, falls and glucocorticosteroid therapy, are presented in Table 1. All the participants had body weight and height measured with bare feet, using standarized weighing and height scales. Based on those measurements, the body mass index (BMI) was calculated as weight (kg)/height (m^2^).

**Table 1:** Demographic data and fracture risk factors required by Garvan and POL-RISK calculators and BMD values

Demographic statistics	Mean (min–max)	Standard deviation (SD)
Age	65.2 (55–87)	6.9
Weight (kg)	74 (40–131)	13.1
Height (cm)	157.9 (143.5–173.5)	5.6
BMI (kg/m^2^)	29.7 (16.2–47.5)	5.1
BMI classification	No. of participants	The percentage of study cohort (**%)**
• Underweight	5	1.3
• Normal weight	65	16.7
• Overweight	150	38.6
• Obesity	169	43.4
Glucocorticosteroid use in a dose of at least 5 mg of prednisone (or equivalent) for 6 weeks or longer	29	7.5
Falls during last 12 months		
• No falls	252	64.6
• 1 fall	65	16.7
• 2 falls	32	8.2
• 3 or more falls	41	10.5
Fracture locations (after the age of 40):	No. of fractures	The percentage of overall fractures (%)
• Forearm	49	55.7
• Ankle	31	35.2
• Lumbar spine	4	4.55
• Hip	4	4.55
Total fracture count	88	
DXA results	Mean (min–max)	Standard deviation (SD)
BMD (g/cm^2^)	0.738 (0.465–1.122)	0.112
*T*-score	−0.99 (−3.5–2.5)	1.05
WHO classification (based on *T*-score)	No. of participants	The percentage of study cohort (%)
• Normal bone density	171	43.8
• Osteopenia	193	49.5
• Osteoporosis	25	6.4

### Densitometry

Dual-energy X-ray absorptiometry measurements were performed, using a Hologic Explorer (Hologic Inc., Waltham, MA, USA; with the software version: 13.0:3). Bone mineral density (areal BMD, g/cm^2^), *T*-score and *Z*-score of non-dominant femoral neck were measured. *T*-and *Z*-scores were calculated on the basis of the National Health and Nutrition Examination Survey (NHANES) database for white women. All the analyses were performed by one experienced technician. Based on the repeated measurements of 25 women, the precision of DXA measurements, expressed as coefficient of variation (CV%), was 2.03%. The DXA results are presented together with demographic data in Table 1.

### Fracture risk assessment

In order to evaluate fracture risk for the subsequent 5 years, the data, from medical history, and DXA results were entered into the following calculators: the Garvan Institute Fracture Risk calculator and the POL-RISK calculator.

The Garvan algorithm takes into account the following clinical factors: gender, patient’s age, the number of fractures since the patient’s age of 50 (excluding major injuries, e.g. after car accidents), the number of falls over the last 12 months and densitometry results (depending on densitometer type). The last factor is not obligatory in fracture risk calculations. The designers of the tool suggest that the results for any fracture risk below 8% in a 5-year perspective enable to assume a low risk of fracture, while the results above 13% correspond to high fracture risk. Any values between 8 and 13% should be considered for individualized pharmacological treatment.

In the POL-RISK algorithm, the following factors are considered: osteoporotic fracture after the age of 40, caused by fall from a height not above the body height and if occurred at the following locations: vertebra, hip, femoral shaft, forearm, arm, lower leg, rib and foot, any falls during the last 12 months, steroid use within the last year in doses of mg of prednisone (or equivalent) for a period of 6 weeks or longer, the actual body height. Both calculators refer to *T*-score values, obtained in femoral neck DXA measurements. In the POL-RISK algorithm, these values are obligatory for further calculations. Cut-off values in POL-RISK model have not yet been set for patients with low and high limits of fracture risk.

As there are some differences, especially in fracture classification (but also in the categorization of falls), the fracture risk was calculated separately in each participant for the period of 5 years, taking into account the above-mentioned inclusion and exclusion criteria. In the Garvan calculator, the 5-year fracture risk was calculated for any osteoporotic/fragility fracture (not for hip fracture separately).

### Statistical analysis

Statistical analyses were performed with the Statistica 13.0 (StatSoft Polska Sp. z o.o. 2020 and STATISTICA Scorecard version 6.0.76) software packages. The normality of data was assessed by histograms and the Shapiro-Wilk test. Descriptive statistics for quantitative variables were presented as mean values and standard deviations and as median and interquartile ranges for non-normal distribution. The correlation analysis was done by the Spearman’s correlation test. The kappa-Cohen’s calculator was used to assess the level of conformity between the two methods. In order to establish optimal thresholds for the POL-RISK algorithm, a receiver operator characteristic (ROC) analysis was performed. Based on the ROC curves and Youden’s index, the sensitivity and specificity for cut-off values were obtained for low-, intermediate- and high-risk groups. A *p* value less than 0.05 was regarded as statistically significant.

## Results

### Fracture risk calculators

The median value of fracture risk, established by the Garvan calculator, was 7% (in the range 1–54%; the interquartile range [IQR] 6%). The number of subjects classified at low (the Garvan value less than 8%), moderate (Garvan 8–13%) and high (Garvan above 13%) fracture risk level were 217 (55.8%), 123 (31.6%) and 49 (12.6%), respectively.

The median value, obtained in the POL-RISK algorithm, was 8.8% (range 1.1–45.5%; IQR 8%).

There was a significant correlation between the results from both compared calculators (*r*=0.6, *p*<0.005).

### Clinical conformity

**Fig. 1:**
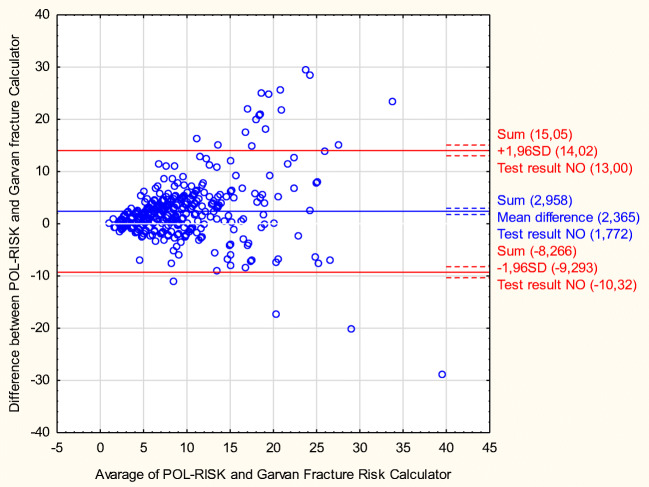
Bland-Altman plot for the concordance between the results from the Garvan Fracture Risk Calculator and the POL-RISK algorithm

In order to establish conformity for the classification of subjects at the assumed fracture risk category, the same threshold values, as presented above for the Garvan calculator results (8 and 13%) were used for the POL-RISK algorithm. Then, the compliance was assessed by the kappa-Cohen’s calculator. For the threshold of 8% conformity was 76.1% (Cohen’s kappa—0.53; moderate concordance). For the threshold of 13%, conformity raised up to 84.1% (Cohen’s kappa—0.46; moderate concordance). The moderate concordance between the Garvan Fracture Risk Calculator and the POL-RISK algorithm was also suggested by the Bland-Altman plot (see Fig. 1).

**Fig. 2:**
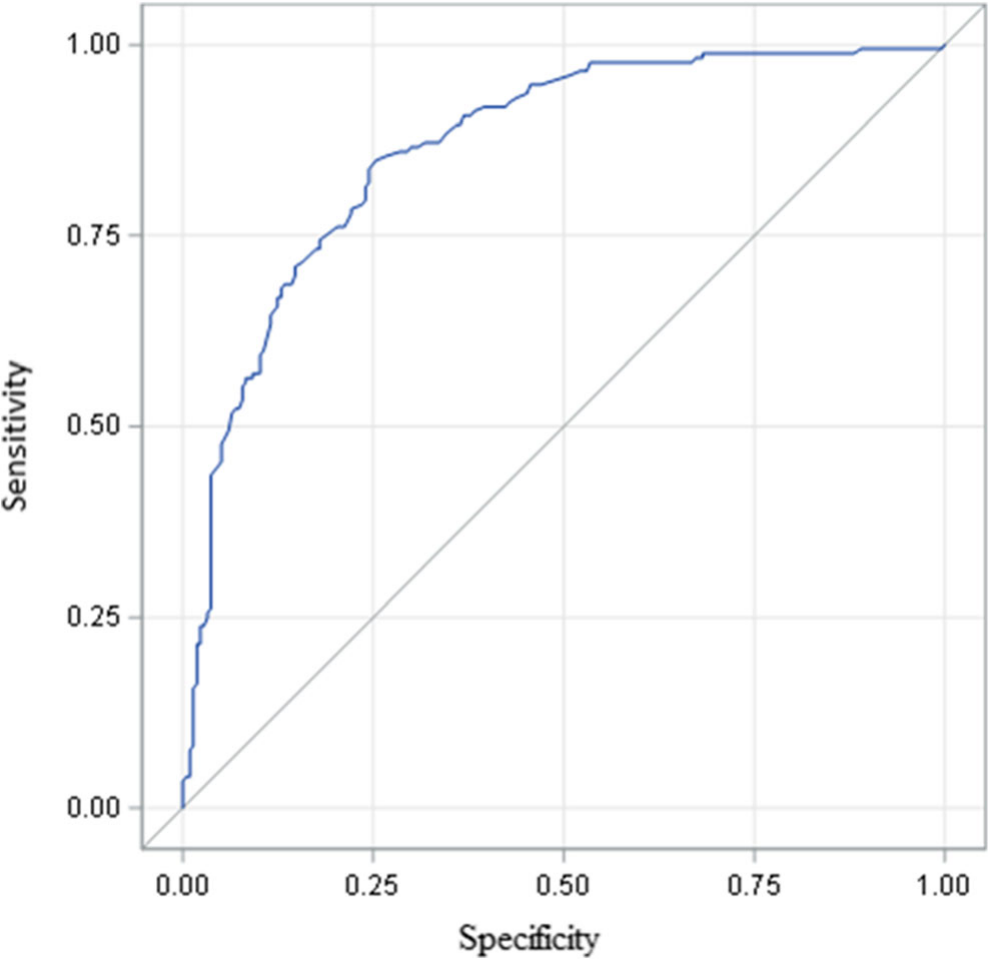
ROC curve for POL-RISK results vs. Garvan calculator results, stratified according to the 8% threshold (AUC=0.865)

**Fig. 3:**
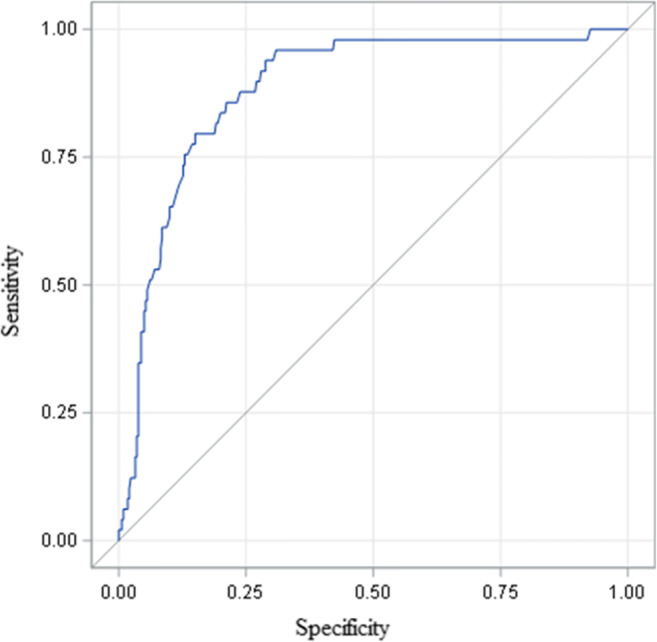
ROC curve for POL-RISK results vs. the Garvan calculator results, stratified according to the 13% threshold (AUC=0.884)

The receiver operating characteristic (ROC) analysis was performed for the POL-RISK algorithm, using the Garvan model as a reference tool with two different cut-offs, assumed as indicators of high fracture risk (see Figs. 2 and 3). For 8% threshold, the area under curve (AUC) was 0.865 (95% confidence interval [CI] 0.829–0.901). For the 13% threshold, the AUC value was 0.884 (95% CI 0.837–0.931).

ROC analysis was also applied in an attempt to establish optimal cut-offs for the POL-RISK algorithm, corresponding to low and high fracture risk, according to the Garvan method. Based on Youden’s Index the threshold in the POL-RISK model, identifying the low-risk group, was less than 8.7%, while it was 11% for the high-risk group (see Tables 2, 3 and 4).

**Table 2:** Sensitivity, specificity and Youden Index for POL-RISK cut-offs, referred to Garvan calculator results, stratified according to the 8% threshold (i.e. low fracture risk threshold by the Garvan method)

Cut-off	Sensitivity	Specificity	Youden Index
8.3	0.867	0.7	0.567
8.4	0.861	0.71	0.566
8.5	0.86	0.714	0.575
8.6	0.855	0.733	0.587
8.7 *	**0.849**	**0.747**	**0.595**
8.8	0.837	0.756	0.593
8.9	0.82	0.756	0.576
9	0.814	0.76	0.574
9.4	0.785	0.779	0.564
9.9	0.744	0.82	0.564

*The optimal (according to Youden Index) POL-RISK threshold, corresponding to the low fracture risk threshold, established for Garvan calculator

**Table 3:** Sensitivity, specificity and Youden Index for POL-RISK cut-offs, referred to Garvan calculator results, stratified according to the 13% threshold (i.e. high fracture risk threshold by the Garvan method)

Cut-off	Sensitivity	Specificity	Youden Index
10.3	0.959	0.685	0.644
10.4	0.959	0.688	0.647
10.5	0.959	0.691	0.65
10.8	0.939	0.703	0.642
10.9	0.939	0.709	0.648
11 *	**0.939**	**0.712**	**0.651**
12.5	0.857	0.785	0.642
12.6	0.857	0.788	0.645
13.7	0.796	0.844	0.64
13.8	0.796	0.85	0.646

*The optimal (according to Youden Index) POL-RISK threshold, corresponding to the high fracture risk threshold, established for the Garvan calculator

**Table 4:** Optimal cut-offs for in the POL-RISK algorithm for low and high fracture risk groups, based on ROC analysis

	POL-RISK cut-offs	
	8.7%	11%
Sensitivity (95% CI; *p*)	0.849 (0.786–0.899; 0.001)	0.939 (0.831–0.987; 0.001)
Specificity (95% CI; *p*)	0.747 (0.683–0.803; 0.001)	0.712 (0.66–0.759; 0.001)
Positive predictive value (95% CI; *p*)	0.726 (0.659–0.787; 0.001)	0.319 (0.244–0.402; 0.001)
Negative predictive value (95% CI; *p*)	0.861 (0.804–0.908; 0.001)	0.988 (0.965–0.998; 0.001)
Accuracy in Garvan and POL-RISK (95% CI; *p*)	0.792 (0.748–0.831; 0.001)	0.704 (0.694–0.783; 0.001)

Based on the proposed thresholds, the study participants were classified into low, intermediate and high fracture risk groups, as presented in Table 5.

**Table 5:** Fracture risk group classification, based on the Garvan Fracture Risk Calculator and the proposed thresholds in the POL-RISK algorithm

	No. of participants:	The percentage of study group (%)
Garvan nomogram		
Low fracture risk (<8%)	217	55.8
Moderate fracture risk (8–13%)	123	31.6
High fracture risk (>13%)	49	12.6
POL-RISK		
Low fracture risk (<8.7%)	188	48.3
Intermediate fracture risk (8.7–11%)	57	14.7
High fracture risk (>11%)	144	37

## Discussion

Both diagnostic tools, i.e. the Garvan Fracture Risk Calculator and the POL-RISK calculator, were compared in the reported study in a long-term, 5-year observation. Despite some differences, regarding risk factors, identified as significant in the used methods, the general conformity between the two methods turned out fairly good. We may consider that the observation was the most important finding in the described study. When using the same threshold values in the POL-RISK algorithm as those in the Garvan calculator, clinical conformity was 76.1–84.1% (Cohen’s kappa 0.46–0.53), which means moderate concordance. After analysing ROCs, we found out the POL-RISK thresholds to have been corresponding to those, proposed by the Garvan Institute, which could then be interpreted by the following decisions: up to 8.7% fracture risk—no indications for pharmacological intervention, 8.7–11%—pharmacological intervention might be considered with individual approach, 11% and higher—pharmacological treatment is necessary. The thresholds, proposed by the designers of the Garvan tool for the 5-year risk of any fracture, were as follows: up to 8%, 8–13%, 13% and more, respectively. It is worth emphasizing that, although both tools use common factors, such as fractures at middle-age or falls, the factors are not dose-dependent in the Polish algorithm. The same is with age which impacts the results in the Garvan algorithm but in the Polish algorithm, it is only an inclusion criterion in the case of women.

Both algorithms use DXA results from the femoral neck but it is not obligatory in the Garvan calculator. Nevertheless, Bolland et al. [21] proved that the Garvan calculator, as well as FRAX, provided worse discrimination without BMD. Similar observations can be found in the study of Holloway-Kew et al., comparing FRAX and Garvan fracture risk calculators in Australian women and men [22]—Garvan results—with no BMD values—were characterized by worse predictability of fracture risk in both genders, whereas FRAX calculator results did not differ, regarding their discriminating ability, either with or without BMD values. Nevertheless, the authors concluded that both calculators underestimated the actual fracture risk. As a continuation of that research, the same authors assessed fracture prediction ability of FRAX after adjusting the Trabecular Bone Score (TBS) but still, the results were similar to those with no TBS adjustment [23]. The limitation of that lumbar spine DXA-derived measurement was its constrained usability in patients on antiresorptive therapy [24]. Moreover, in order to calculate TBS, a commercially available software is needed [25]. The availability of densitometers is still rather low in many regions and the available software in those devices is questionable. Summing up, BMD is still the best-known and reliable bone quality index and its use should be obligatory in every case when pharmacological intervention is considered.

Falls seem to be the most common risk factor for bone fractures—it is taken into account in both tools analysed in the reported study, but not in the FRAX calculator. It is estimated that about 30–40% of elderly patients may fall at least once a year [26, 27]. This prognosis is consistent with our study in which 35% of participants reported at least one fall during the last 12 months. There may be plenty of causes of physical falls—postural hypotension, dizziness, impaired balance, muscle weakness, polypharmacy, visual impairment, urinary incontinence. One-third of “fallers” will experience fracture(s) which may lead to serious health complications, loss of independence and increased mortality rates [27–29]. It was already proven that recurrent falls are independent risk factors of fractures and should be taken into account in an overall fracture risk analysis [30–33]. Already in 2011, the International Society for Clinical Densitometry and International Osteoporosis Foundation on FRAX publicized their official position, regarding the history of falls—it was recommended to recognize patients with higher risk of falls and to consider it in the decision-making mechanism, additionally to FRAX risk factors. According to the authors, it was impossible to incorporate this risk factor into the FRAX calculator [34].

The Garvan calculator helps predict the fracture risk in the perspective of 5 and 10 years, whereas the Polish algorithm, based on the prospective, still on-going RAC-OST-POL study, predicts fracture risk only in a 5-year perspective. The FRAX calculator predicts fracture risk during a 10-year period. Regarding middle-aged women, such a long perspective is useful for clinical decision-making; the problem appears in senile patients with life expectancy less than 10 years. In such patients, the risk calculators with the 5-year prediction perspective seem to be more appropriate. In this group of patients, pharmacological treatment is recommended if the fracture risk is high but the therapy must be carefully tailored to the general health condition of the patient. It is documented that a combined osteoporosis therapy may reduce the risk of fracture (vertebral and probably hip) even more than in younger groups [35, 36].

The clinical concordance between the Garvan Fracture Risk Calculator and the POL-RISK algorithm demonstrated with use of Bland-Altman plot suggested quite good conformity within low and moderate values with marked variance with higher values of mean scores. Nevertheless, the amount of those is small and needs observation on bigger group.

The study limitation was the lack of a similar algorithm for the Polish population during a 10-year observation period which will soon be available though. Only women were observed and so, fracture risks for males were not established. The fracture statistics was based on patients’ reports with no possibility to see spine X-ray pictures or to have access to past medical records for verification. In this way, some silent vertebral fractures could have been omitted and the number of any fractures in the spine could have been underestimated. However, both methods, based on long-term observation periods of postmenopausal women, did generate comparable results. To the best knowledge of the authors, the reported study was the first one which attempted to compare the POL-RISK algorithm with the Garvan calculator, regarding the fracture risk prediction performance of both tools. The study group consisted of postmenopausal women only which ensured a higher homogeneity level of the studied population.

## Conclusions

The conformity of results of the compared POL-RISK and Garvan calculator methods was fairly good, based on low- and high-risk cut-off values, established in ROC analysis. The optimal thresholds, proposed to identify subjects at moderate fracture risk, are 8.7–11% and at high risk over 11%.

## Data Availability

2015

## Code availability

The database and all calculations were done with the use of Statistica 13.0 software (StatSoft Polska Sp. z o.o. 2020 and STATISTICA Scorecard version 6.0.76).
